# High-Capacity and Long-Lifespan Aqueous LiV_3_O_8_/Zn Battery Using Zn/Li Hybrid Electrolyte

**DOI:** 10.3390/nano11061429

**Published:** 2021-05-28

**Authors:** Qiang Pang, Xiangyu Yu, Shijing Zhang, Wei He, Siyu Yang, Yao Fu, Ying Tian, Mingming Xing, Xixian Luo

**Affiliations:** 1 School of Science, Dalian Maritime University, Dalian 116026, China; xiangyuyu@dlmu.edu.cn (X.Y.); zhangshijing@dlmu.edu.cn (S.Z.); weihe@dlmu.edu.cn (W.H.); 1120190826@dlmu.edu.cn (S.Y.); tianying@dlmu.edu.cn (Y.T.); mingmingxing@dlmu.edu.cn (M.X.); luoxx@dlmu.edu.cn (X.L.); 2 College of Chemistry, Jilin University, Changchun 130012, China

**Keywords:** aqueous battery, cathode material, hybrid electrolyte, LiV_3_O_8_, Zn-ion battery

## Abstract

Aqueous zinc-ion batteries (AZIBs) are promising candidates for large-scale energy storage because of their low cost and high safety. However, their practical applications are impeded by low energy density and short service life. Here, an aqueous Zn^2+^/Li^+^ hybrid-ion battery is fabricated using the LiV_3_O_8_ nanorods as the cathode, metallic Zn as the anode, and 3 M Zn(OTf)_2_ + 0.5 M LiOTf aqueous solution as the electrolyte. Compared with the batteries using pure 3 M Zn(OTf)_2_ electrolyte, the cycle performance of the hybrid-ion battery is significantly improved. After 4000 cycles at 5 A g^1^, the remaining capacity is 163.9 mA h g^−1^ with impressive capacity retention of 87.0%. Ex-situ XRD, ex-situ XPS, and SEM tests demonstrate that the hybrid electrolyte can inhibit the formation of the irreversible Zn_3_(OH)_2_V_2_O_7_·2H_2_O by-product and restrict Zn dendrite growth during cycling, thereby improving the cycle performance of the batteries.

## 1. Introduction

The harm caused by the unreasonable use of fossil energies and environmental pollutions urge us to develop and utilize renewable energies (such as solar energy, wind energy, and tidal energy) with the objective of electricity generation. However, due to the dispersion of these energy sources in time and geographic locations, there is an urgent need to develop a low-cost and reliable electrical energy storage technology to integrate these energies [1,2,3]. Lithium-ion batteries, as the most successful consumer batteries, are not applicable to large-scale energy storage systems mainly due to their high cost and low safety [4,5,6]. As one of the candidates for the next generation of rechargeable batteries, aqueous zinc-ion batteries (AZIBs) are considered very promising in large-scale energy storage because of their unique advantages [7]. Metallic zinc can be used as the anode electrode for AZIBs. It can provide a proper negative potential of −0.762 V vs. standard hydrogen electrode (SHE) and provide a high capacity of ~820 mA h g^−1^. However, the cathode electrodes face some complex challenges, such as limited capacity and unsatisfactory cycle life caused by poor electrochemical reaction kinetics and severe side reactions, limiting the practical applications of AZIBs [8].

Recently, many kinds of materials have been reported as cathode electrodes for AZIBs, including manganese-based oxides [9,10,11], vanadium-based oxides [12,13,14,15], phosphates [16,17], Prussian blue analogues [18,19,20], organic compounds [21,22,23], etc. Among them, vanadium-based oxides show great potential as cathode materials for high-performance AZIBs due to their suitable operation voltage and high capacity. However, the vanadium-based cathodes suffer from the dissolution of vanadium ions and the structural degradation during the cycling process in aqueous electrolytes, resulting in poor cyclic stability [24]. Therefore, many intelligent strategies have been proposed to improve the performance of vanadium-based cathode materials. For instance, Mai et al. [25] prepared a V_2_O_5_·H_2_O/graphene composite as the cathode electrode for AZIBs. This composite electrode showed a better rate performance and longer cycle life than the sample without crystal water. Structure and spectroscopy characterizations demonstrated that the high electronic conductivity of graphene and the shielding effect of water molecules jointly improved the reversibility of Zn^2+^ insertion/extraction into/from V_2_O_5_ and promoted the diffusion kinetics of Zn^2+^ ions Recent studies [26,27] demonstrate that adjusting the electrolyte composition is also an effective method for improving battery performance. Zhang et al. [28] reported that pre-adding magnesium ions into the electrolyte could provide an appropriate equilibrium balance between the dissolution and recombination of magnesium vanadate, thus leading to high cyclic stability of the Mg_x_V_2_O_5_·nH_2_O cathode in AZIBs.

Among the many vanadium-based oxides, LiV_3_O_8_ has been highlighted as a promising cathode material for rechargeable batteries due to its unique layered structure and high theoretical capacity of up to 560 mA h g^−1^ based on the conversion between V^5+^ and V^3+^ [29]. He et al. [30] illustrated a reversible V^5+^/V^3+^ double redox with the phase transformation between α-Zn_x_LiV_3_O_8_ (x < 2) and β-Zn_y_LiV_3_O_8_ (2 ≤ y ≤ 3). Benefiting from this multi-electron transition mechanism, the LiV_3_O_8_ cathode delivered a high capacity of 557.5 mA h g^−1^ at the current density of 10 mA g^−1^. Kim et al. [31] studied the detailed phase evolution of LiV_3_O_8_ during Zn intercalation using electrochemistry, in-situ XRD, and simulation techniques. A reversible transformation between a stoichiometric ZnLiV_3_O_8_ phase and a solid-solution Zn_y_LiV_3_O_8_ phase was revealed, which was different from that observed in lithium-ion batteries. However, a low average capacity (~180 mA h g^−1^) in the initial cycles and a fast capacity decay of 75% retention of the maximum capacity after 65 cycles were reported. Therefore, there is an urgent need to fully utilize the theoretical capacity of LiV_3_O_8_ for AZIBs while achieving high cyclic stability.

In this work, LiV_3_O_8_ was synthesized by a sol-gel method, and its layered structure and nanorod morphology were confirmed by XRD, SEM, and TEM tests. Aqueous Zn^2+^/Li^+^ hybrid-ion batteries were assembled using the LiV_3_O_8_ nanorods as the cathode, metallic Zn as the anode, and 3 M Zn(OTf)_2_ + 0.5 M LiOTf aqueous solution as the electrolyte. Compared with the batteries using 3 M Zn(OTf)_2_ electrolyte, the cycle performance of the hybrid-ion battery was significantly improved. At a current density of 0.1 A g^−1^, the battery provided a high discharge capacity of 389.8 mA h g^−1^. After 4000 cycles at 5 A g^−1^, the remaining capacity was 163.9 mA h g^−1^ with an impressive capacity retention of 87.0%. Ex-situ XPS and ex-situ XRD tests showed that the hybrid electrolyte could inhibit the formation of by-product during cycling, leading to the improved cycle performance of the aqueous LiV_3_O_8_/Zn batteries.

## 2. Materials and Methods

### 2.1. Preparation of LiV_3_O_8_

The LiV_3_O_8_ sample was prepared by a sol-gel method. In a typical synthesis, 0.702 g NH_4_VO_3_ (99% A.R., Sinopharm Chemical Reagent Co., Ltd., Beijing, China) and 1.008 g oxalic acid dihydrate (99.9% A.R., Sinopharm Chemical Reagent Co., Ltd., Beijing, China) were added into 36 mL deionized water at 70 °C and stirred for 30 min. Then, 0.084 g LiOH (99.9% A.R., Sinopharm Chemical Reagent Co., Ltd., Beijing, China) was added into the solution, and it was stirred at 70 °C for 90 min to obtain a homogenous solution. Then, the mixture was stirred at around 80 °C to dry. The obtained precursor was well ground and calcined at 400 °C for 2 h in an air atmosphere with a heating rate of 5 °C min^−1^. The final product was obtained after cooling down to room temperature.

### 2.2. Material Characterizations

The structural analysis of the LiV_3_O_8_ powder was performed by X-ray diffraction (XRD) using an XRD-6000X X-ray diffractometer (Shimadzu Corporation, Kyoto, Japan) with Cu K_α_ radiation. The XRD tests of the electrodes were carried out using a D/MAX-Ultima X-ray diffractometer with Co K_α_ radiation. The samples’ morphological properties and element mapping were observed using scanning electron microscopy (SEM) on a SUPRA 55 SAPPHIRE field-emission microscope (Carl Zeiss Corporation, Jena, Germany) equipped with an energy dispersive spectrometer (EDS). Transmission electron microscopy (TEM) and high-resolution transmission electron microscopy (HRTEM) were performed on a JOEL JEM-2100 transmission electron microscope (Joel Corporation, Tokyo, Japan). X-ray photoelectron spectroscopy (XPS) measurements were performed on a Thermo ESCALAB 250Xi instrument (Shimadzu Corporation, Kyoto, Japan) configured with a monochromatic Al K_α_ (1486.6 eV) to analyze the surface chemical states of the samples. The cells after charge or discharge were disassembled in the air. The electrodes were thoroughly washed with deionized water, dried in an oven at 70 °C, and then used for XRD, SEM, and XPS testes.

### 2.3. Electrochemical Experiments

The working (cathode) electrode was prepared by mixing 60 wt% LiV_3_O_8_ sample, 30 wt.% acetylene black (super P, SAIBO Electrochemical Material Co., Ltd., Shenzhen, China), and 10 wt% polyvinylidene difluoride (PVDF, SAIBO Electrochemical Material Co., Ltd., Shenzhen, China) in N-methyl pyrrolidone (NMP, 99%, SAIBO Electrochemical Material Co., Ltd., Shenzhen, China) solvent. The well-mixed slurry was coated on a stainless-steel foil current collector and dried at 60 °C for 12 h. Then, the electrode was cut into circles with a diameter of 0.8 cm. The loading density was about 1 mg cm^−2^. The two electrolytes were 3 M Zn(CF_3_SO_3_)_2_ and 3 M Zn(CF_3_SO_3_)_2_ + 0.5 M LiCF_3_SO_3_ aqueous solutions, respectively. Zn(CF_3_SO_3_)_2_ (Zn(OTf)_2_) and LiCF_3_SO_3_ (LiOTf) were purchased from Aladdin Biochemical Technology Co., Ltd. (Shanghai, China). The pH values of 3 M Zn(CF_3_SO_3_)_2_ and 3 M Zn(CF_3_SO_3_)_2_ + 0.5 M Li(CF_3_SO_3_) electrolytes are 3.13 (±0.01) and 3.08 (±0.01), respectively. The counter (anode) electrode was zinc metal, which was polished with sandpaper before use. The working and counter electrodes were separated by a Whatman GF/C glass fiber filter. The cells (CR2032 coin-type) were assembled in the air and their electrochemical properties were tested using a LAND CT2001 battery tester at room temperature. All the batteries in this work were charged and discharged at a constant current mode. The voltage range is 0.4–1.6 V vs. Zn^2+^/Zn. The applied constant current was calculated based on the mass of the active material multiplied by the current density. For cycling stability tests, both the charge and discharge current densities are 0.1 A g^−1^, 2.0 A, or 5.0 A g^−1^. For rate capability tests, both the charge and discharge current densities are 0.1, 0.3, 0.5, 1.0, 2.0, and 5.0 A g^−1^. As the highest capacity of ~400 mA h g^−1^ was achieved at a moderate current density of 0.1 A g^−1^, a current density of 400 mA g^−1^ was defined as 1C. The potential stability window of the electrolytes was characterized by cyclic voltammetry (CV) between −0.2 and 2.7 V (vs. Zn^2+^/Zn) using an electrochemical workstation (CHI 636E). The potential scan rate is 1 mV s^−1^. In the CV test, a stainless-steel foil was used as the working electrode, and Zn foil was used as both the reference and counter electrodes. The actual pictures of the experimental set-up (Figure S1) and the experimental uncertainty analysis can be found in the Supporting Information.

## 3. Results and Discussion

**Figure 1:**
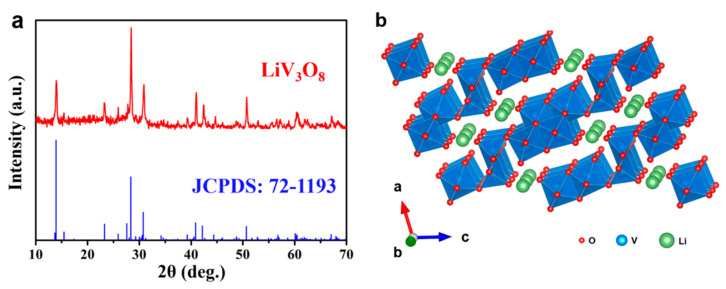
(**a**) XRD pattern and (**b**) structure model of LiV_3_O_8_.

**Figure 2:**
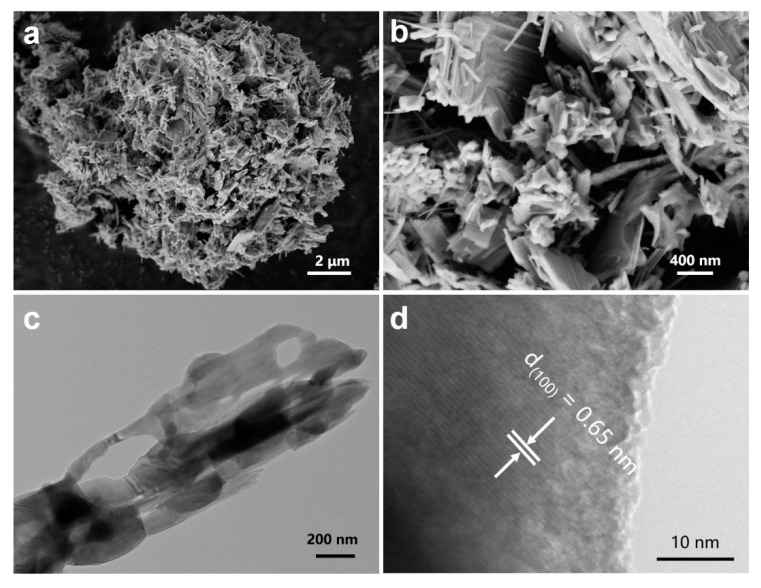
(**a**,**b**) SEM, (**c**) TEM, and (**d**) HRTEM images of LiV_3_O_8_.

Figure 1a shows the XRD pattern of the prepared sample. All diffraction peaks of the sample correspond well to the monoclinic LiV_3_O_8_ (JCPDS No.: 01-072-1193) with the *P*2_1_/*m* space group (a = 6.68 Å, b = 3.60 Å, c = 12.03 Å). The strong and sharp diffraction peaks indicate the high crystallinity of the sample, and no extra peaks belonging to impurities are found. As shown in Figure 1b, a LiV_3_O_8_ unit cell is composed of two VO_6_ octahedrons and a VO_5_ tetrahedron, forming a V_3_O_8_ layer along the (100) plane and these layers are connected by Li^+^ ions [30]. This unique layered structure can facilitate the insertion/extraction of foreign ions. The micro-morphology of the as-prepared LiV_3_O_8_ sample was observed by SEM. The sample particles show irregular spherical morphology with a diameter of about tens of micrometers (Figure S2). V and O elements are distributed uniformly in the sample particle (Figure S3). The spherical particles are assembled from irregular nanorods, and the length and width of the nanorods are several micrometers (Figure 2a,b). TEM and HRTEM characterizations were further conducted to detect the morphology and structure of the sample. Some nanopores can be found on a LiV_3_O_8_ nanorod (Figure 2c). The HRTEM image (Figure 2d) clearly shows a set of lattice fringes, which indicates that the sample has high crystallinity. The lattice spacing of 0.65 nm corresponds to the (100) planes, which is in accordance with the analysis of the XRD pattern.

**Figure 3:**
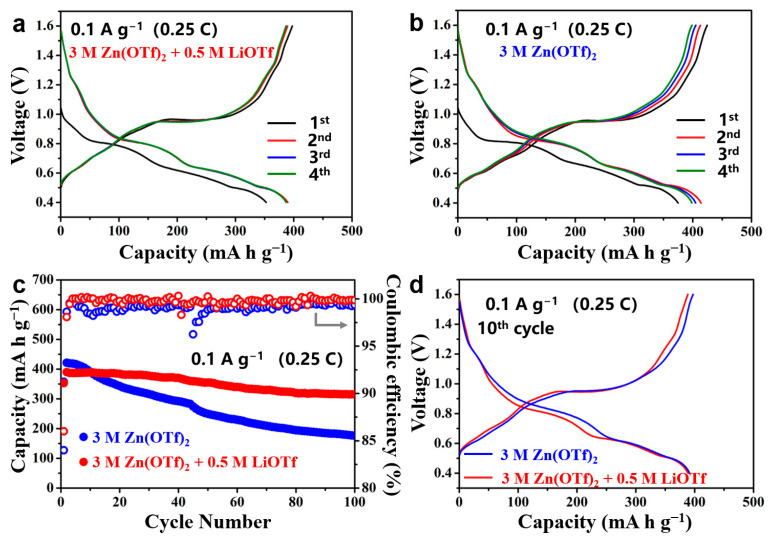
Charge and discharge profiles of the cells using (**a**) 3 M Zn(OTf)_2_ + 0.5 M LiOTf electrolyte and (**b**) 3 M Zn(OTf)_2_ electrolyte, respectively; (**c**) cycle performances and coulombic efficiencies of the cells at 0.1 A g^−1^ (0.25C); (**d**) charge and discharge profiles of the cells at the 10th cycle.

The potential stability window of the electrolytes was characterized by CV at a potential scan rate of 1 mV s^−1^ between −0.2 and 2.7 V vs. Zn^2+^/Zn. A stainless-steel foil was used as the working electrode, and Zn foil was used as both reference and counter electrodes. As shown in Figure S4, the potential stability window of the two electrolytes is almost the same (0–2.0 V). Therefore, there are not water side reactions occurring during cycling within a narrow voltage range of 0.4–1.6 V vs. Zn^2+^/Zn. The electrochemical properties of the as-prepared LiV_3_O_8_ for AZIBs were then investigated using 3 M Zn(OTf)_2_ + 0.5 M LiOTf hybrid electrolyte and 3 M Zn(OTf)_2_ electrolyte, respectively, within a voltage range of 0.4–1.6 V vs. Zn^2+^/Zn. Figure 3a shows the first four galvanostatic charge-discharge profiles of the battery using the hybrid electrolyte at a current density of 0.1 A g^−1^ (0.25C). The initial discharge capacity is 352.8 mA h g^−1^, and the following charge capacity is 397.5 mA h g^−1^, resulting in an initial coulombic efficiency of 88.8%. In the subsequent second cycle, a higher discharge capacity of 389.8 mA h g^−1^ is achieved. It can be seen that the lower first discharge capacity is caused by the lack of a small discharge plateau at around 1.25 V. The open circle voltage of the newly assembled cells is below 1.2 V. Therefore, during the first discharge, the battery cannot display this discharge plateau, resulting in a lower discharge capacity. In the subsequent cycles, four charge–discharge voltage plateaus can be found. It indicates that the charge/discharge process involves a multistep redox reaction. As reported by He et al., the Zn insertion process can be divided into three stages. In the first discharge stage (1.6–0.9 V), a solid-solution single-phase reaction (from LiV_3_O_8_ to Zn_0.5_LiV_3_O_8_) occurs. In the subsequent discharge process (0.9–0.55 V), the discharge profile shows two consecutive discharge voltage plateaus, which are caused by a two-phase transformation process from Zn_0.5_LiV_3_O_8_ to ZnLiV_3_O_8_ and from ZnLiV_3_O_8_ to Zn_2_LiV_3_O_8_. In the last discharge stage, the voltage plateau between 0.55 and 0.4 V is attributed to a phase transition from Zn_2_LiV_3_O_8_ to Zn_3_LiV_3_O_8_. In the following charge process, the structural evolutions are almost reversed. It is evident that the charge-discharge profiles of the hybrid battery, except for the first discharge, are almost overlapped. It indicates that the electrochemical reactions are highly reversible. The battery using 3 M Zn(OTf)_2_ electrolyte shows similar charge-discharge profiles to that using the hybrid electrolyte. Although the former exhibits a higher initial discharge capacity of 375.2 mA h g^−1^, a fast capacity loss in the following cycles is observed. Besides, the initial coulombic efficiency of this battery (87.8%) is lower than that of the hybrid battery, suggesting that more side reactions may occur in the battery using 3 M Zn(OTf)_2_ electrolyte. The cycling stability of the batteries was then investigated at a current density of 0.1 A g^−1^. As shown in Figure 3c, although the battery using 3 M Zn(OTf)_2_ electrolyte shows a higher initial capacity, its remaining capacity after 100 cycles is only 176 mA h g^−1^ with a capacity retention of 41.8%. In contrast, a high reversible capacity of 315.2 mA h g^−1^ has remained after 100 cycles for the hybrid battery, and the capacity retention is 80.8%. More importantly, the calculated average coulombic efficiency of the hybrid battery in 100 cycles reaches up to 99.6%, which is higher than that of the battery using 3 M Zn(OTf)_2_ electrolyte (98.9%). The higher coulombic efficiency of the hybrid battery demonstrates that adding extra Li salt into the electrolyte can improve the reversibility of the electrochemical reactions, leading to better cycling stability. Figure 3d displays the 10th charge-discharge profiles of the two batteries. It can be seen that the charge–discharge profile shape and the positions of the voltage plateaus of these two batteries are almost the same. It suggests that the LiV_3_O_8_ cathode has the same reaction mechanism in the two different electrolytes, and Li^+^ ions may not, at least not largely, participate in the Zn^2+^ insertion/extraction processes during cycling. After 100 cycles (Figure S5), the voltage plateaus’ length of the battery using the 3 M Zn(OTf)_2_ electrolyte shrinks, which suggests that the capacity decay is due to the loss of active material. As shown in Figure S6, the cycling stability of the hybrid batteries was performed before and after removing the oxygen. The oxygen was removed by passing argon through the electrolyte for 1 h. It can be found that the influence of oxygen on the capacity and cycling stability is negligent.

**Figure 4:**
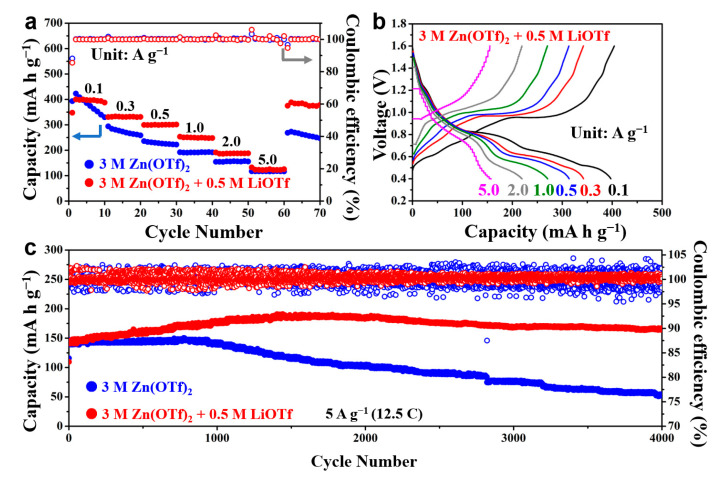
(**a**) Rate capability of the two batteries at different current densities; (**b**) galvanostatic charge and discharge profiles of the hybrid battery at different current densities; (**c**) long-cycle stability of the two batteries at 5.0 A g^−1^.

The rate and long-cycle performance were then tested to evaluate the rapid charge/discharge capability and lifespan of the batteries. Figure 4a shows the two batteries’ specific discharge capacity and coulombic efficiency at different current densities of 0.1 (0.25C), 0.3 (0.75C), 0.5 (1.25C), 1.0 (2.5C), 2.0 (5C), and 5.0 A g^−1^ (12.5C). It can be seen that the battery using 3 M Zn(OTf)_2_ electrolyte delivers a higher capacity than the hybrid battery in the initial cycles but undergoes a fast capacity at low current densities. When the current densities are increased to 1.0, 2.0, and 5.0 A g^−1^, the specific capacity does not decrease significantly and reaches 192.1, 154.3, and 117.0 mA h g^−1^, respectively. In comparison, the hybrid battery gives higher capacities of 400.1, 330.7, 299.5, 253.1, 191.2, and 132.5 mA h g^−1^ at 0.1, 0.3, 0.5, 1.0, 2.0, and 5.0 A g^−1^, respectively. In addition, when the current density is recovered from 5.0 A g^−1^ to 0.1 A g^−1^, the specific capacity of the battery using 3 M Zn(OTf)_2_ electrolyte is 268.4 mA h g^−1^. In contrast, the hybrid battery can still deliver a reversible capacity of 375.1 mA h g^−1^, which is close to the initial capacity, indicating that the addition of Li^+^ ions into the electrolyte can enhance the rate capability and durability of the batteries. Figure 4b shows the charge and discharge curves of the hybrid battery at different current densities. The shape of the curves does not change significantly and shows no obvious polarization with the current density increasing. It reveals that the LiV_3_O_8_ electrode has good electrochemical reaction kinetics in the hybrid electrolyte. It is interesting that the battery using the 3 M Zn(OTf)_2_ electrolyte shows better capacity retention at high rates. It can be found that the discharge voltage plateau below 0.6 V of the battery using the pure electrolyte almost disappears when the current density is higher than 1.0 A g^−1^ due to the large polarization (Figure S7). Even though the current density continues to rise, the high voltage plateau at about 0.8 V can maintain very well. This leads to an improved capacity retention of the battery without Li^+^. However, the voltage plateau below 0.6 V of the hybrid battery just disappears when the current density is increased to 5.0 A g^−1^. This causes a distinct capacity reduction in the hybrid battery. The long-cycle performances of the batteries at a current density of 5 A g^−1^ (12.5C) are shown in Figure 4c. In the beginning, the two batteries show similar specific discharge capacities. The capacity of the battery using the 3 M Zn(OTf)_2_ electrolyte begins to decrease dramatically after about 800 cycles, and only shows a capacity of 53.9 mA h g^−1^ after 4000 cycles. The capacity retention rate is only 36.7% to the highest capacity. The capacity of the hybrid battery experiences a rise from the initial 144.6 mA h g^−1^ to the highest capacity of 188.4 mA h g^−1^, and then begins to decay slowly. The capacity increase in the initial cycles is probably attributed to the electrolyte penetration and the electrochemical activation process, which is commonly observed in vanadium-based electrode materials for AZIBs [27,31,32,33,34]. After 4000 cycles, there is still a remaining capacity of 163.9 mA h g^−1^, and the capacity retention rate is 87.0% relative to the highest capacity. The final capacity is even higher than the initial capacity. To sum up, the LiV_3_O_8_ cathode shows a high capacity close to 400 mA h g^−1^, and its cycling stability is remarkably improved by using a Zn^2+^/Li^+^ hybrid electrolyte, demonstrating great potential for the high-performance aqueous zinc-based battery.

**Figure 5:**
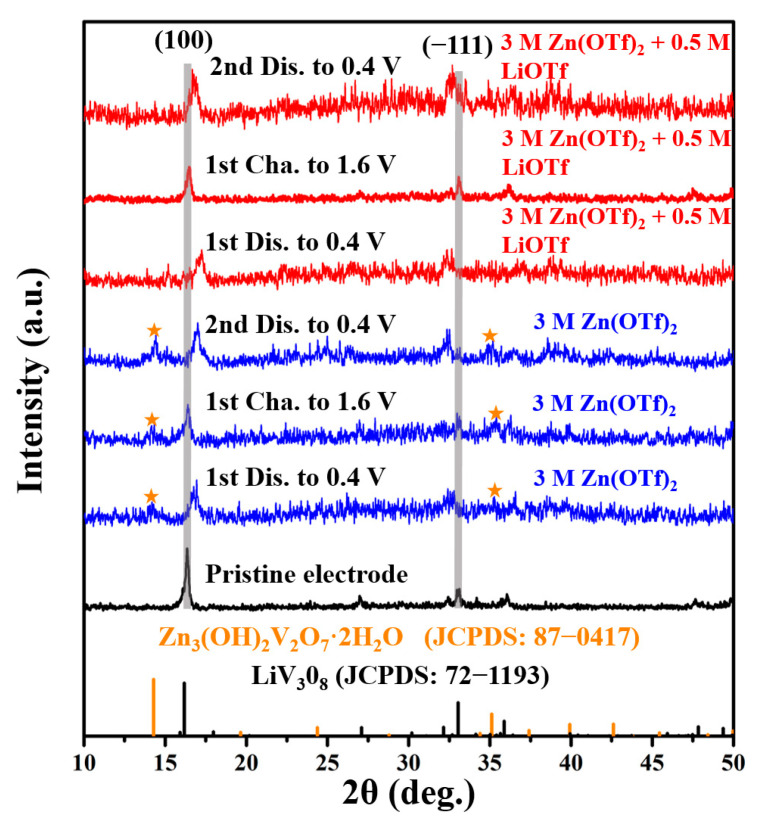
Ex-situ XRD patterns of the LiV_3_O_8_ electrodes at different charge/discharge states.

To further explore the functions of Li^+^ ions in the electrolyte, ex-situ XRD patterns of the two batteries at different charging/discharging stages were analyzed. As shown in Figure 5, the XRD peaks of the pristine electrode correspond well to the monoclinic LiV_3_O_8_. After the first and second discharges, the characteristic peak at 16.2°, which corresponds to the (100) plane, shifts toward a higher diffraction angle. It indicates that the lattice spacing of the (100) plane is reduced due to the stronger electrostatic interactions in V_3_O_8_ layers caused by Zn^2+^ intercalation [35]. Moreover, the characteristic peaks at 33.0° corresponding to the (−111) plane shift to a lower diffraction angle after discharging, which indicates that the interlayer spacing of the (−111) planes of LiV_3_O_8_ in two batteries is enlarged. After charging, all the diffraction peaks belonging to LiV_3_O_8_ move to the original positions in the two batteries, proving the same reaction mechanism of the LiV_3_O_8_ host in different electrolytes. It is worth noting that two new diffraction peaks are found at 14.3° and 35.1° in the XRD patterns of the battery using 3 M Zn(OTf)_2_ electrolyte after discharging and charging. Besides, the intensity of these two peaks increases after the second discharge. It demonstrates that a new irreversible phase forms during cycling, and its amount increases as the number of cycles increases. By searching the PDF standard card library, it is known that the new phase is Zn_3_(OH)_2_V_2_O_7_·2H_2_O, which usually has no electrochemical activity [36]. In sharp contrast, no extra XRD peaks are found in the XRD patterns of the hybrid battery. It demonstrates that adding Li^+^ ions into the electrolyte can suppress the formation of Zn_3_(OH)_2_V_2_O_7_·2H_2_O by-product in aqueous LiV_3_O_8_/Zn batteries, thus largely preventing the loss of active material and improving the cycling stability of the batteries.

**Figure 6:**
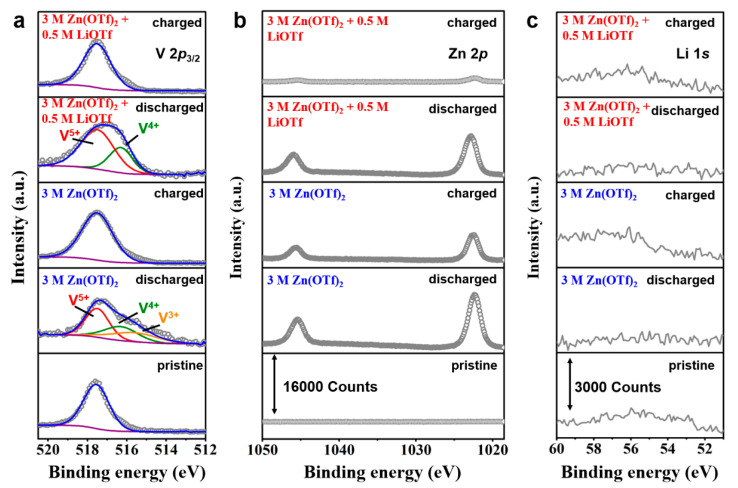
(**a**) V 2p, (**b**) Zn 2p, and (**c**) Li 1s XPS regions of the LV_3_O_8_ electrodes in different electrolytes at different charge/discharge states.

**Figure 7:**
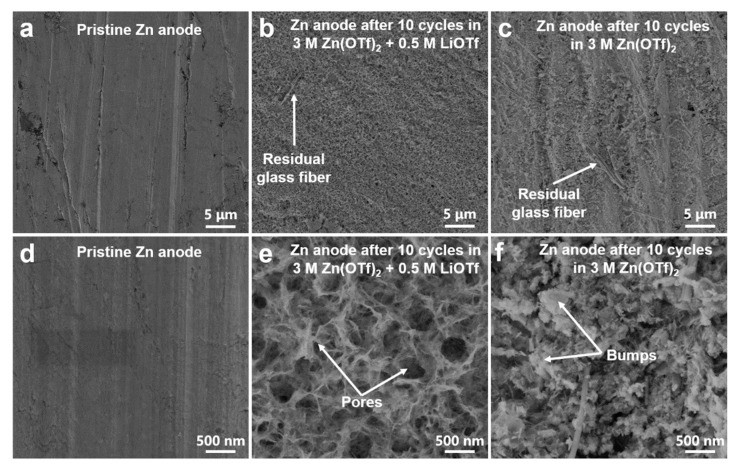
SEM images of (**a**,**d**) the fress pristine Zn anode, (**b**,**e**) the Zn anode after 10 cycles in 3 M Zn(OTf)_2_ + 0.5 M LiOTf, and (**c**,**f**) the Zn anode after 10 cycles in 3 M Zn(OTf)_2_ + 0.5 M LiOTf.

As shown in Figure 6, ex-situ XPS tests were carried out to explore the evolution of chemical speciation of the electrodes during the first charging/discharging processes. Figure 6a shows the V 2p_3/2_ regions of the cathodes. In the pristine electrode, the peak at 517.5 eV is attributed to the V^5+^ in LiV_3_O_8_ [37] The appearance of V^4+^ in the discharged hybrid battery demonstrates the electrochemical reduction of V element by the intercalation of Zn^2+^ ions [38] A deeper reduction from V^5+^ to V^3+^ is found in the discharged LiV_3_O_8_ electrode using the 3 M Zn(OTf)_2_ electrolyte, which is in accordance with its higher initial capacity than the hybrid battery. The reduction of V is entirely reversed in the charging process, where V^5+^ is regenerated. As shown in Figure 6b, there is no Zn XPS signal in the pristine electrode. Two strong signal peaks of Zn 2p at 1045.0 eV and 1021.9 eV are present in the discharged electrodes for the two different batteries, which prove the intercalation of Zn^2+^ in two electrodes. [39] In the following charging process, the Zn signal intensity of the electrode in the hybrid electrolyte is significantly decreased, confirming the extraction of the most Zn^2+^ from the LiV_3_O_8_ host. It is worth noting that a strong Zn signal is detected on the charged electrode in 3 M Zn(OTf)_2_ electrolyte, indicating the poor reversibility of Zn^2+^ intercalation/extraction. According to the ex-situ XRD analysis, this is due to the formation of Zn_3_(OH)_2_V_2_O_7_·2H_2_O by-product on the electrode surface. In the pristine electrode, a weak Li 1s signal can be detected at 56 eV. However, there are no obvious Li 1s signal changes in two different batteries upon charge or discharge. It indicates that Li^+^ does not participate in the electrochemical reaction in two different batteries. However, the existence of Li^+^ may influence the electrolyte/electrode interfacial environment and suppress the formation of Zn_3_(OH)_2_V_2_O_7_·2H_2_O by-product. To better understand the influence of Li^+^ ions on Zn dendrite growth, SEM images of the Zn anodes in two different electrolytes before and after 10 cycles at 1.0 A g^−1^ were observed. As shown in Figure 7a,b, the surface of a fresh pristine Zn anode is clean and smooth. After 10 cycles in the 3 M Zn(CF_3_SO_3_)_2_ + 0.5 M Li(CF_3_SO_3_) electrolyte (Figure 7b,e), the surface of the Zn anode shows a honeycomb structure with many irregular porous structures. In contrast, the surface of the Zn anode in the 3 M Zn(CF_3_SO_3_)_2_ electrolyte (Figure 7c,f) is covered by bumps (about tens to hundreds of nanometers in size), which is probably due to the uneven Zn striping/plating. These results prove that the addition of Li^+^ ions into the electrolyte can facilitate the Zn striping/plating process and avoid the growth of Zn dendrite. Therefore, the excellent electrochemical performance of the hybrid batteries is also attributed to the restriction of Zn dendrite growth through the addition of Li^+^ ions.

## 4. Conclusions

In summary, a Zn/Li hybrid electrolyte (3 M Zn(OTf)_2_ + 0.5 M LiOTf aqueous solution) was employed for aqueous LiV_3_O_8_/Zn batteries for the first time. Compared with the pure 3 M Zn(OTf)_2_ electrolyte, the hybrid electrolyte can enable the LiV_3_O_8_/Zn batteries to show a high reversible capacity, enhanced rate performance and durability, and better long-cycle stability. Ex-situ XPS, ex-situ XRD, and SEM characterizations demonstrate that the addition of LiOTf in the electrolyte can effectively inhibit the formation of the Zn_3_(OH)_2_V_2_O_7_·2H_2_O by-product and restrict Zn dendrite growth during cycling, thereby improving the electrochemical performance of the batteries.

## Data Availability

Not applicable.

## Supplementary Materials

The following are available online at https://www.mdpi.com/article/10.3390/nano11061429/s1, Table S1: Definitions of nomenclature, acronyms, and abbreviations, experimental uncertainty analysis, Figure S1. Experimental setup for galvanostatic charge/discharge and CV tests, Figure S2. SEM image of the LiV_3_O_8_ particles, Figure S3. EDS elemental mapping images of LiV_3_O_8_. (a) SEM image of LiV_3_O_8_, (b) V element, (c) O element, Figure S4. Cyclic voltammograms of Zn electrodes in 3 M Zn(CF_3_SO_3_)_2_ electrolyte (blue line) and 3 M Zn(OTf)_2_ + 0.5 M LiOTf electrolyte (red line) at the scan rate of 1 mV s^−1^ between −0.2 and 2.7 V vs. Zn^2+^/Zn, Figure S5. Charge and discharge profiles of the two LiV_3_O_8_/Zn batteries at the 100th cycle at a current density of 0.1 A g^−1^, Figure S6. Cycling stability of the hybrid batteries before and after removing oxygen at the current density of 2 A g^−1^, Figure S7. Charge and discharge profiles of the batteries using the 3 M Zn(CF_3_SO_3_)_2_ electrolyte at different current densities.

## References

[1] Karden E., Ploumen S., Fricke B., Miller T., Snyder K. (2007). Energy storage devices for future hybrid electric vehicles. J. Power Sources.

[2] Tran M.K., Bhatti A., Vrolyk R., Wong D., Panchal S., Fowler M., Fraser R. (2021). A Review of Range Extenders in Battery Electric Vehicles: Current Progress and Future Perspectives. World Electr. Veh. J..

[3] Panchal S. (2016). Experimental Investigation and Modeling of Lithium-Ion Battery Cells and Packs for Electric Vehicles. Ph.D. Thesis.

[4] Tarascon J.M., Armand M. (2001). Issues and challenges facing rechargeable lithium batteries. Nature.

[5] Akhoundzadeh M.H., Panchal S., Samadani E., Raahemifar K., Fowler M., Fraser R. (2021). Investigation and simulation of electric train utilizing hydrogen fuel cell and lithium-ion battery. Sustain. Energy Technol. Assess..

[6] Panchal S. (2014). Impact of Vehicle Charge and Discharge Cycles on the Thermal Characteristics of Lithium-ion Batteries. Master’s Thesis.

[7] Xu C.J., Li B.H., Du H.D., Kang F.Y. (2012). Energetic Zinc Ion Chemistry: The Rechargeable Zinc Ion Battery. Angew. Chem. Int. Ed. Engl..

[8] Tang B.Y., Shan L.T., Liang S.Q., Zhou J. (2019). Issues and opportunities facing aqueous zinc-ion batteries. Energy Environ. Sci..

[9] Wu B.K., Zhang G.B., Yan M.Y., Xiong T.F., He P., He L., Xu X. (2018). Graphene Scroll-Coated α-MnO_2_ Nanowires as High-Performance Cathode Materials for Aqueous Zn-Ion Battery. Small.

[10] Jiang B.Z., Xu C.J., Wu C.L., Dong L.B., Li J., Kang F.Y. (2017). Manganese Sesquioxide as Cathode Material for Multivalent Zinc Ion Batteries with High Capacity and Long Cycle Life. Electrochim. Acta.

[11] Zhang N., Cheng F.Y., Liu Y.C., Zhao Q., Lei K.X., Chen C.C., Liu X.S., Chen J. (2016). Cation-Deficient Spinel ZnMn_2_O_4_ Cathode in Zn(CF_3_SO_3_)_2_ Electrolyte for Rechargeable Aqueous Zn-Ion Batteries. J. Am. Chem. Soc..

[12] Kundu D., Adams B.D., Duffort V., Vajargah S.H., Nazar L.F. (2016). A high-capacity and long-life aqueous rechargeable zinc battery using a metal oxide intercalation cathode. Nat. Energy.

[13] Pang Q., Sun C.L., Yu Y.H., Zhao K.N., Zhang Z.Y., Voyles P.M., Chen G., Wei Y.J., Wang X.D. (2018). H_2_V_3_O_8_ Nanowire/Graphene Electrodes for Aqueous Rechargeable Zinc Ion Batteries with High Rate Capability and Large Capacity. Adv. Energy Mater..

[14] Pang Q., He W., Zhao H.N., Yu X.Y., Wei Y.J., Tian Y., Xing M.M., Fu Y., Luo X.X. (2020). Hierarchical Aluminum Vanadate Microspheres with Structural Water: High-Performance Cathode Materials for Aqueous Rechargeable Zinc Batteries. ChemElectroChem.

[15] Liu X.Y., Ma L.W., Du Y.H., Lu Q.Q., Yang A.K., Wang X.Y. (2021). Vanadium Pentoxide Nanofibers/Carbon Nanotubes Hybrid Film for High-Performance Aqueous Zinc-Ion Batteries. Nanomaterials.

[16] Hu P., Zhu T., Wang X.P., Zhou X.F., Wei X.J., Yao X.H., Luo W., Shi C.W., Owusu K.A., Zhou L. (2019). Aqueous Zn//Zn(CF_3_SO_3_)_2_//Na_3_V_2_(PO_4_)_3_ batteries with simultaneous Zn^2+^/Na^+^ intercalation/de-intercalation. Nano Energy.

[17] Wang F., Hu E.Y., Sun W., Gao T., Ji X., Fan X.L., Han F.D., Yang X.Q., Xu K., Wang C.S. (2018). A rechargeable aqueous Zn^2+^-battery with high power density and a long cycle-life. Energy Environ. Sci..

[18] Zhang L.Y., Chen L., Zhou X.F., Liu Z.P. (2015). Towards High-Voltage Aqueous Metal-Ion Batteries Beyond 1.5 V: The Zinc/Zinc Hexacyanoferrate System. Adv. Energy Mater..

[19] Jia Z.J., Wang B.G., Wang Y. (2015). Copper hexacyanoferrate with a well-defined open framework as a positive electrode for aqueous zinc ion batteries. Mater. Chem. Phys..

[20] Trócoli R., Mantia F.L. (2015). An Aqueous Zinc-Ion Batteries Based on Copper Hexacyanoferrate. ChemSusChem.

[21] Zhao Q., Huang W.W., Luo Z.Q., Liu L.J., Lu Y., Li Y.X., Li L., Hu J.Y., Ma H., Chen J. (2018). High-capacity aqueous zinc batteries using sustainable quinone electrodes. Sci. Adv..

[22] Kundu D., Oberholzer P., Glaros C., Bouzid A., Tervoort E., Pasquarello A., Niederberger M. (2018). Organic Cathode for Aqueous Zn-Ion Batteries: Taming a Unique Phase Evolution toward Stable Electrochemical Cycling. Chem. Mater..

[23] Gou Z.W., Ma Y.Y., Dong X.L., Huang J.H., Wang Y.G., Xia Y.Y. (2018). An Environmentally Friendly and Flexible Aqueous Zinc Battery Using an Organic Cathode. Angew. Chem. Int. Ed..

[24] Wang F., Niu Z.Q. (2019). Design Strategies of Vanadium-based Aqueous Zinc-Ion Batteries. Angew. Chem. Int. Ed..

[25] Yan M.Y., He P., Chen Y., Wang S.Y., Wei Q.L., Zhao K.N., Xu X., An Q.Y., Shuang Y., Shao Y.Y. (2018). Water-Lubricated Intercalation in V_2_O_5_·nH_2_O for High-Capacity and High-Rate Aqueous Rechargeable Zinc Batteries. Adv. Mater..

[26] Wan F., Zhang Y., Zhang L.L., Liu D.B., Wang C.D., Song L., Niu Z.Q., Chen J. (2019). Reversible Oxygen Redox Chemistry in Aqueous Zinc-Ion Batteries. Angew. Chem. Int. Ed..

[27] Wan F., Zhang L.L., Dai X., Wang X.Y., Niu Z.Q., Chen J. (2018). Aqueous Rechargeable Zinc/Sodium Vanadate Batteries with Enhanced Performance from Simultaneous Insertion of Dual Carriers. Nat. Commun..

[28] Zhang Y.M., Li H.N., Huang S.Z., Fan S., Sun L.G., Tian B.B., Chen F.M., Wang Y., Shi Y.M., Yang H.Y. (2020). Rechargeable Aqueous Zinc-Ion Batteries in MgSO_4_/ZnSO_4_ Hybrid Electrolytes. Nano-Micro Lett..

[29] Ohzuku T., Ueda A. (1994). Why transition metal (di)oxides are the most attractive materials for batteries. Solid State Ion..

[30] He P., Yan M.Y., Liao X.B., Luo Y.Z., Mai L.Q., Nan C.W. (2020). Reversible V^3+^/V^5+^ double redox in lithium vanadium oxide cathode for zinc storage. Energy Storage Mater..

[31] Xie Z.Q., Lai J.W., Zhu X.P., Wang Y. (2018). Green Synthesis of Vanadate Nanobelts at Room Temperature for Superior Aqueous Rechargeable Zinc-Ion Batteries. ACS Appl. Energy Mater..

[32] Liu C.F., Neale Z., Zheng J.Q., Jia X.X., Huang J.J., Yan M.Y., Tian M., Wang M.S., Yang J.H., Cao G.Z. (2019). Expanded hydrated vanadate for high-performance aqueous zinc-ion batteries. Energy Environ. Sci..

[33] Ming F.W., Liang H.F., Lei Y.J., Kandambeth S., Eddaoudi M., Alshareef H.N. (2018). Layered Mg_x_V_2_O_5_·nH_2_O as Cathode Material for High Performance Aqueous Zinc Ion Batteries. ACS Energy Lett..

[34] Zhang N., Dong Y., Jia M., Bian X., Wang Y.Y., Qiu M.D., Xu J.Z., Liu Y.C., Jiao L.F., Cheng F.Y. (2018). Rechargeable Aqueous Zn-V_2_O_5_ Battery with High Energy Density and Long Cycle Life. ACS Energy Lett..

[35] Alfaruqi M.H., Mathew V., Song J.J., Kim S., Islam S., Pham D.T., Jo J., Kim S., Baboo J.P., Xiu Z.L. (2017). Electrochemical Zinc Intercalation in Lithium Vanadium Oxide: A High-Capacity Zinc-Ion Battery Cathode. Chem. Mater..

[36] Pan Z.H., Yang J., Yang J., Zhang Q.C., Zhang H., Li X., Kou Z.K., Zhang Y.F., Chen H., Yan C.L. (2020). Stitching of Zn_3_(OH)_2_V_2_O_7_·2H_2_O 2D Nanosheets by 1D Carbon Nanotubes Boosts Ultrahigh Rate for Wearable Quasi-Solid-State Zinc-Ion Batteries. ACS Nano.

[37] Zhan W.X., Fan C.L., Zhang W.H., Yi G.D., Chen H., Han S.C., Liu J.S. (2020). Ultra-long cycle life and high-rate performance subglobose Na_3_V_2_(PO_4_)_2_F_3_@C cathode and its regulation. Int. J. Energy Res..

[38] Liu F., Chen Z.X., Fang G.Z., Wang Z.Q., Cai Y.S., Tang B., Zhou J., Liang S.Q. (2019). V_2_O_5_ nanospheres with mixed vanadium valences as high electrochemically active aqueous zinc-ion battery cathode. Nano-Micro Lett..

[39] Pang Q., He W., Yu X.Y., Yang S.Y., Zhao H.N., Fu Y., Xing M.M., Tian Y., Luo X.X., Wei Y.J. (2021). Aluminium pre-intercalated orthorhombic V_2_O_5_ as high-performance cathode material for aqueous zinc-ion batteries. Appl. Surf. Sci..

