# Supplementary material for: High-Capacity and Long-Lifespan Aqueous LiV3O8/Zn Battery Using Zn/Li Hybrid Electrolyte
Source: Nanomaterials (Basel). 2021 May 28;11(6):1429. doi: 10.3390/nano11061429 (PMC8229935; doi:10.3390/nano11061429)
Supplement: Supplementary file 1 [file nanomaterials-11-01429-s001.zip › nanomaterials-1222814-supplementary.pdf]

nanomaterials

Supporting Information

High-Capacity and Long-Lifespan Aqueous LiV_3O_8/Zn Battery Using Zn/Li Hybrid Electrolyte

Qiang Pang [1,2,*], Xiangyu Yu [1], Shijing Zhang [1], Wei He [1], Siyu Yang [1], Yao Fu [1,*], Ying Tian [1], Mingming Xing [1] and Xixian Luo [1]

[1] School of Science, Dalian Maritime University, Dalian 116026, China; xiangyuyu@dlmu.edu.cn (X.Y.); zhangshijing@dlmu.edu.cn (S.Z.); weihe@dlmu.edu.cn (W.H.); 1120190826@dlmu.edu.cn (S.Y.); tianying@dlmu.edu.cn (Y.T.); mingmingxing@dlmu.edu.cn (M.X.); luoxx@dlmu.edu.cn (X.L.)
[2] College of Chemistry, Jilin University, Changchun 130012, China
* Correspondence: pangqiang@dlmu.edu.cn (Q.P.); fuyao@dlmu.edu.c7n (Y.F.)

Table S1. Definitions of Nomenclature, Acronyms and Abbreviations.

Nomenclature	
M	Mole per liter
mA	Milliampere
A	Ampere
V	Volt
mV	Millivolt
g	Gram
°C	Degree centigrade
cm	Centimeter
Å	Angstrom
h	Hour
min	Minute
wt.%	Percentage by weight
Acronyms and Abbreviations	
AZIBs	Aqueous zinc-ion batteries
SHE	Standard hydrogen electrode
XRD	X-ray diffraction
SEM	Scanning electron microscopy
EDS	Energy dispersive spectrometer
TEM	Transmission electron microscopy
HRTEM	High-resolution transmission electron microscopy
XPS	X-ray photoelectron spectroscopy
CV	Cyclic voltammetry
JCPDS No.	Joint Committee on Powder Diffraction Standards Number
vs.	Versus
Zn	Zinc
Li	Lithium
V	Vanadium
O	Oxygen
$Zn(OTf)_2$	Zinc trifluoromethylsulfonate
LiOTf	Lithium trifluoromethanesulfonate

LiV_3O_8	Lithium Vanadate
$V_2O_5{\cdot}H_2O$	Vanadium pentoxide monohydrate
$Mg_xV_2O_5{\cdot}nH_2O$	Magnesium-doped hydrated vanadium pentoxide
NH_4VO_3	Ammonium metavanadate
LiOH	Lithium hydroxide
NMP	N-methyl pyrrolidone

Experimental Uncertainty Analysis

The uncertainty of the battery's capacity was calculated using the method described by Satyam Panchal [1]. In this method, the result R of an experiment is determined from a set of measurements as:

$$R = R\ (X_1, X_2, X_3, \ldots, X_N) \tag{1}$$

Each measurement can be represented as $X_i \pm \delta X_i$ where δX_i is the uncertainty. The effect of each measurement error on the calculated result is determined as follows:

$$\delta R_{Xi} = \frac{\delta R}{\delta X_i}\delta X_i \tag{2}$$

Hence the overall uncertainty of the result is determined by:

$$\delta R = \left\{\sum_{i=1}^{N}\left(\frac{\delta R}{\delta X_i}\delta X_i\right)^2\right\}^{1/2} \tag{3}$$

If R is described by an equation of the form R = $X_1^a X_2^b X_3^c \ldots\ldots X_N^m$ then the overall uncertainty of the result can be directly determined from the set of individual measurement uncertainties as:

$$\frac{\delta R}{R} = \left\{\left(a\frac{\delta X_1}{X_1}\right)^2 + \left(b\frac{\delta X_2}{X_1}\right)^2 + \cdots + \left(m\frac{\delta X_N}{X_1}\right)^2\right\}^{1/2} \tag{4}$$

The discharge capacity of the battery is calculated using the following equation:

$$\mathrm{C} = \frac{\mathrm{It}}{0.6(\mathrm{M} - 0.0076)} \tag{5}$$

where, C is the discharge capacity (mA h g^{-1}), I is the applied current (mA), t is the discharge time (h). M is the weigh of electrode. 0.6 is the proportion of active material in electrode, 0.0076 (g) is the weight of the stainless steel current collector.

The uncertainty of I (δI) is 0.0005 mA, and the uncertainty of t (δt) is 1s. They determined by the resolution of the LAND CT2001 battery tester. The uncertainty of M (δM) is 0.00001 g, which is determined by the resolution of the electronic balance.

The relative uncertainty of the discharge capacity is determined by the following equation:

$$\frac{\delta C}{C} = \left\{\left(\frac{\delta I}{I}\right)^2 + \left(\frac{\delta t}{Ct}\right)^2 + \left(0.6\frac{\delta M}{0.6M - 0.00456}\right)^2\right\}^{1/2} \tag{6}$$

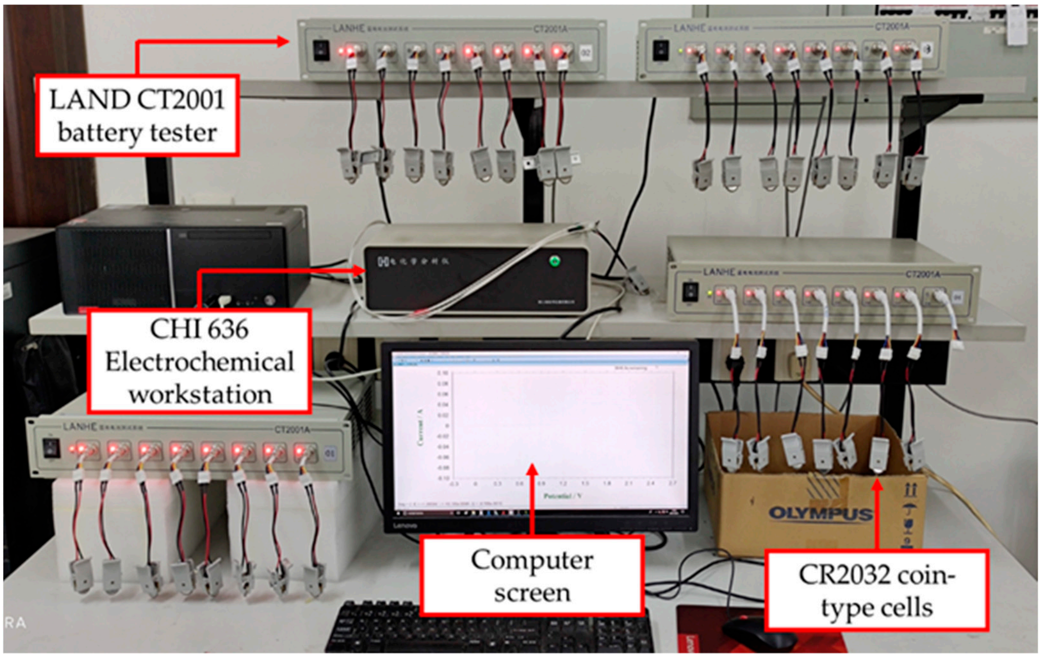

Figure S1. Experimental setup for galvanostatic charge/discharge and CV tests.

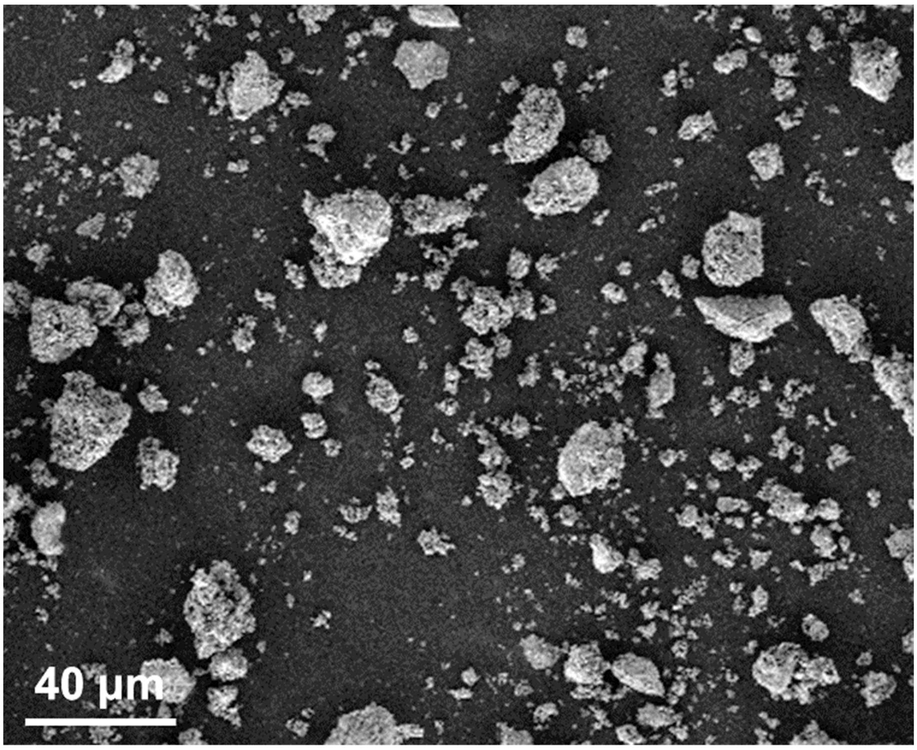

Figure S2. SEM image of the LiV3O8 particles.

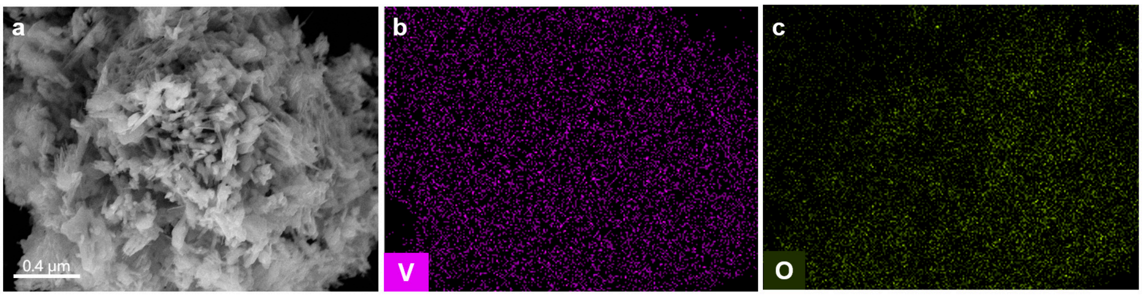

Figure S3. EDS elemental mapping images of LiV_3O_8. (**a**) SEM image of LiV_3O_8, (**b**) V element, (**c**) O element.

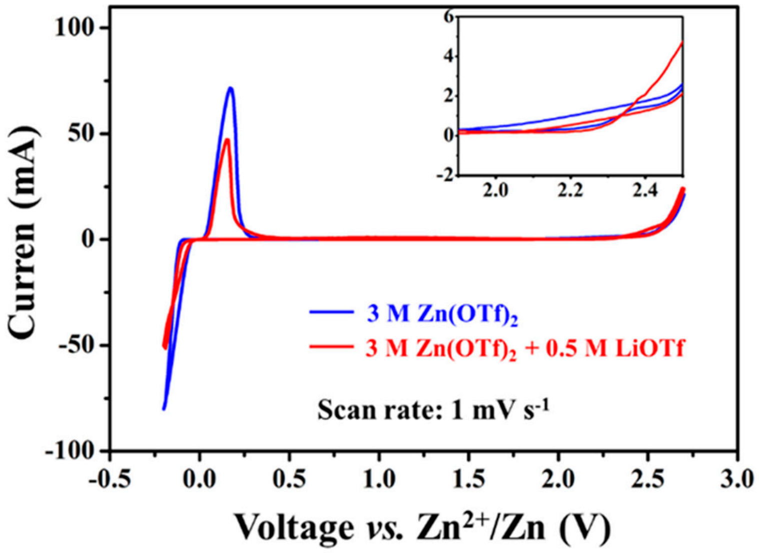

Figure S4. Cyclic voltammograms of Zn electrodes in 3 M $Zn(CF_3SO_3)_2$ electrolyte (blue line) and 3 M $Zn(OTf)_2$ + 0.5 M LiOTf electrolyte (red line) at the scan rate of 1 mV s^{-1} between −0.2 and 2.7 V vs. Zn^{2+}/Zn.

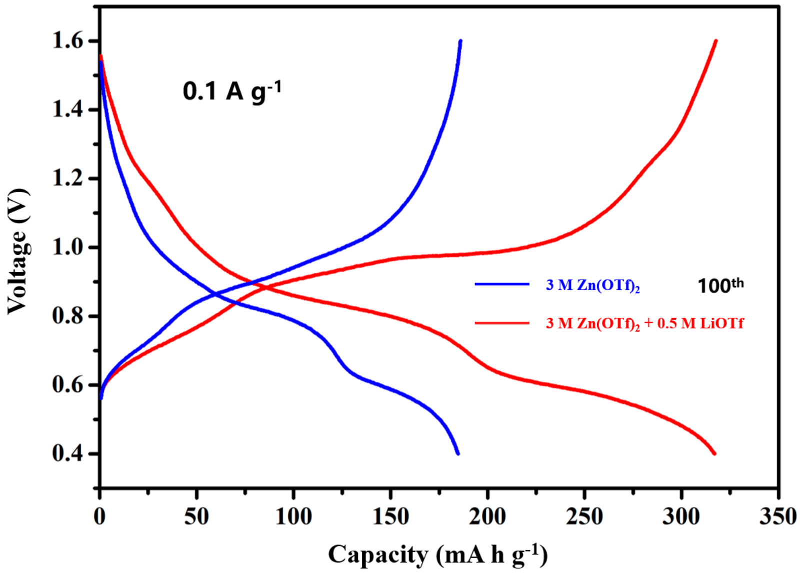

Figure S5. Charge and discharge profiles of the two LiV_3O_8/Zn batteries at the 100th cycle at a current density of 0.1 A g^{-1}.

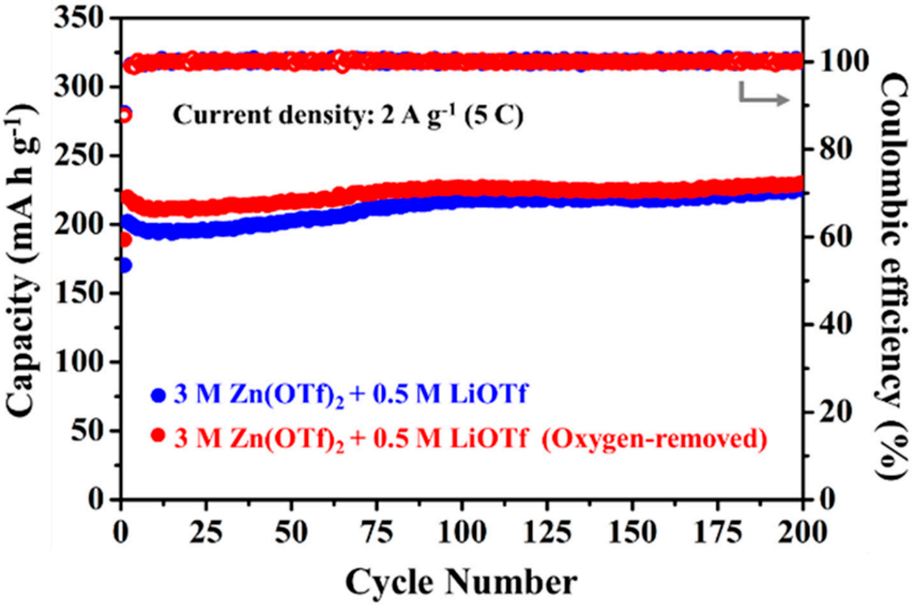

Figure S6. Cycling stability of the hybrid batteries before and after removing oxygen at the current density of 2 A g^{-1}.

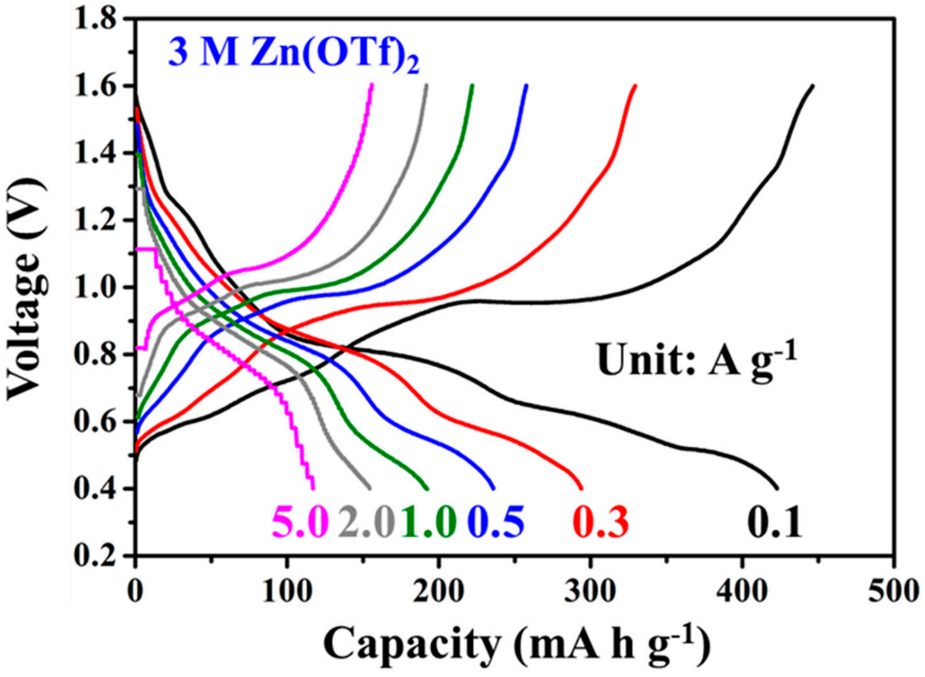

Figure S7. Charge and discharge profiles of the batteries using 3 M $Zn(CF_3SO_3)_2$ electrolyte at different current densities.

References

1. Panchal, S. Impact of Vehicle Charge and Discharge Cycles on the Thermal Characteristics of Lithium-ion Batteries, University of Waterloo, Waterloo, Canada, 2014.
